# Unraveling cardiac anomalies in pediatric neurofibromatosis type 1: insights and implications

**DOI:** 10.1007/s00431-025-06702-8

**Published:** 2026-01-15

**Authors:** Sohier Yahia, Zahraa Abdelmoneim, Dina Ghozzy, Yahya Wahba, Hany M. Abo-Haded

**Affiliations:** 1 Pediatrics Department, Faculty of Medicine, Mansoura University, Mansoura, Egypt; 2 Genetics and Metabolic Unit, Mansoura University Children’s Hospital, Faculty of Medicine, Mansoura University, Mansoura, Egypt; 3 Cardiology Unit, Mansoura University Children’s Hospital, Faculty of Medicine, Mansoura University, Mansoura, Egypt

**Keywords:** Neurofibromatosis type 1, Echocardiography, Myocardial Dysfunction

## Abstract

Neurofibromatosis type 1 (NF1) is an autosomal dominant syndrome caused by mutations in the NF1 gene. Although cardiac abnormalities have been observed in NF1, they are frequently overlooked due to a lack of routine cardiac surveillance. Myocardial strain imaging offers a sensitive and non-invasive method for detecting early subclinical myocardial dysfunction. This study aims to detect cardiac abnormalities in children with NF1 using conventional echocardiography, Doppler tissue imaging (DTI), and myocardial strain analysis. A case–control study was conducted on 38 asymptomatic children with clinically confirmed NF1 and 35 healthy, age- and sex-matched controls. All patients underwent ECG, conventional echocardiography, DTI, and two-dimensional speckle-tracking echocardiography. NF1 patients showed significantly decreased ejection fraction (*p* = 0.0009) and higher interventricular septal and posterior wall thickness during systole (*p* < 0.0001). DTI revealed reduced mitral systolic (Sm) and early diastolic (Em) velocities, longer isovolumic contraction and relaxation periods, and increased LV Tei index values (*p* < 0.0001), indicating combined systolic and diastolic dysfunction. Also, myocardial strain analysis in NF1 children revealed considerably lower peak systolic left ventricular global longitudinal strain (LVGLS) (*p* 0.0014), as well as lower peak systolic septal and lateral wall strain values (*p* 0.0046, 0.0027), respectively.

*Conclusion*: Children with NF1 show early subclinical myocardial dysfunction, even when there is no hypertension or overt cardiac symptoms. These findings highlight the significance of frequent echocardiographic screening, including strain imaging, for the early diagnosis and longitudinal monitoring of heart function in NF1 children.

**Table:** 

**What is Known:** • *Neurofibromatosis type 1 (NF1) is a multisystem syndrome that can involve the cardiovascular system.* • *Previous studies showed hypertrophic cardiac changes in NF1 patients, but data in children, especially those without hypertension, are limited, as routine echocardiography is not involved in NF1 management.*
**What is New:** • *Our study revealed early subclinical myocardial dysfunction in NF1 children without the presence of hypertension or overt cardiac symptoms.* • *This emphasizes the potential of myocardial strain imaging as a sensitive tool for early detection of myocardial dysfunction in NF1 children, thereby supporting the need for routine echocardiographic surveillance in these patients.*

**What is Known:**

• *Neurofibromatosis type 1 (NF1) is a multisystem syndrome that can involve the cardiovascular system.*

• *Previous studies showed hypertrophic cardiac changes in NF1 patients, but data in children, especially those without hypertension, are limited, as routine echocardiography is not involved in NF1 management.*

**What is New:**

• *Our study revealed early subclinical myocardial dysfunction in NF1 children without the presence of hypertension or overt cardiac symptoms.*

• *This emphasizes the potential of myocardial strain imaging as a sensitive tool for early detection of myocardial dysfunction in NF1 children, thereby supporting the need for routine echocardiographic surveillance in these patients.*

## Introduction

Neurofibromatosis 1 (NF1) is one of the neurocutaneous syndromes affecting approximately one in every 2699 births. It follows an autosomal dominant inheritance pattern characterized by complete penetrance, variable phenotypic expression, and a high spontaneous mutation rate [1]. It impacts the nervous system, among other organs and tissues. NF1 is produced by a genetic variant in the neurofibromin gene, found on chromosome 17q11.2. The neurofibromin protein belongs to the GTPase-activating protein family and functions as a tumor suppressor [2]. Therefore, individuals with NF1 are at a higher risk of developing cancers such as peripheral nerve sheath tumor, leukemia, and rhabdomyosarcoma [3].

Penetrance beyond childhood is nearly complete. The clinical phenotype varies significantly, and there are links between the genotype and phenotype [4, 5].

Confirmatory testing for the NF1 variation is not necessary for patients who meet diagnostic criteria [6]. NF1 syndrome can affect multiple systems, necessitating a multidisciplinary approach for accurate diagnosis and appropriate treatment [7].

There is limited research on the prevalence of cardiovascular abnormalities in NF1 patients, including congenital heart disease (CHD), vasculopathy, and hypertension [4]. Previous data estimate that up to 27% of individuals have a cardiovascular aberration of various types [8].

NF1 pertains to a range of several monogenic syndromes, currently classed together as the RASopathies, caused by germline mutations in genes encoding (rat sarcoma/mitogen-activated protein kinase) Ras/MAPK pathway components. Although traditionally separate, they exhibit overlapping phenotypic traits such as facial dysmorphism, cardiac, cutaneous, musculoskeletal, gastrointestinal, and ophthalmic problems, as well as a risk of cancer [9].

One of these syndromes is Noonan syndrome, where pulmonary valve stenosis and hypertrophic cardiomyopathy (HCM) are common cardiac abnormalities in [10]. Similarly, cardiomyopathies have been reported in NF1, occurring with or without accompanying hypertension [11, 12].

Since only a tiny portion of individuals with NF1 syndrome have a routine echocardiography assessment, the reported frequency of cardiac anomalies in these patients is probably underestimated. Myocardial strain imaging is a noninvasive echocardiographic method that measures global and localized myocardial dysfunction to identify subclinical heart illness [13]. Myocardial strain measurements are now considered additional evidence for early detection of myocardial injury, even with maintained fractional shortening or ejection fraction [14, 15].

There are limited studies on myocardial deformation in patients with NF1, with or without hypertension. This study aims to assess the presence of cardiac abnormalities and myocardial dysfunction using conventional echocardiographic, Doppler tissue imaging, and myocardial strain imaging.

## Subjects and methods

### Study design and participants

Case–control study includes consecutive asymptomatic children with NF1 aged below 18 years who visit our neurofibromatosis tertiary outpatient genetics clinics in Mansoura University Children’s Hospital (MUCH) from November 2024 to February 2025. The diagnosis of NF1 was made according to the clinical criteria reported by the National Institutes of Health Consensus Statement Conference [6].

Healthy children were recruited from the outpatient clinic at MUCH as a control group.

Patients were excluded if they had clinical heart failure, a history of cardiovascular disease, chronic renal insufficiency, or other genetic or metabolic diseases, or if they had a known history of hypertension, hyperlipidaemia, or diabetes, or if they were using vasoactive drugs, lipid-lowering drugs, antihypertensive, or antidiabetic treatments.

### Sample size

The sample size calculation was done by G*Power 3.1.9.2. (Universität Kiel, Germany). According to a previous study, the mean ± SD of global longitudinal strain in NF-1 patients was − 19.3 ± 1.7 mm and in healthy controls was − 21.5 ± 2.7 mm [16]. The sample size was based on the following considerations: 0.975 effect size, 95% confidence level, 95% power of the study, and a group ratio of 1:1. The sample size is 29 in each group; we recruited 35 children in each group to account for potential dropouts.

## Cardiac assessment

Cardiac evaluation was performed at the Pediatric Cardiology Unit. Thirty-eight patients with NF1 underwent a complete physical examination, blood pressure measurement, a 12-lead electrocardiography (ECG), and detailed echocardiography. As a control group, we included ECG and echocardiographic studies of 35 healthy age and sex matched children who were routinely referred for echocardiographic evaluation for other screening purposes.

### Conventional echocardiography

Transthoracic echocardiography was performed by an experienced pediatric cardiologist who was blinded to the clinical findings and past medical history of the subjects.

Echocardiographic evaluation was performed using standard pediatric imaging protocols in accordance with American Society of Echocardiography guidelines. Parameters assessed included left ventricular dimensions (interventricular septum thickness, LV posterior wall thickness, LV internal diameter diastolic thickness, and LV internal diameter systolic thickness) and systolic function indices (fractional shortening and ejection fraction).

### Pulsed‑wave Doppler tissue imaging (DTI) studies

**Fig. 1:**
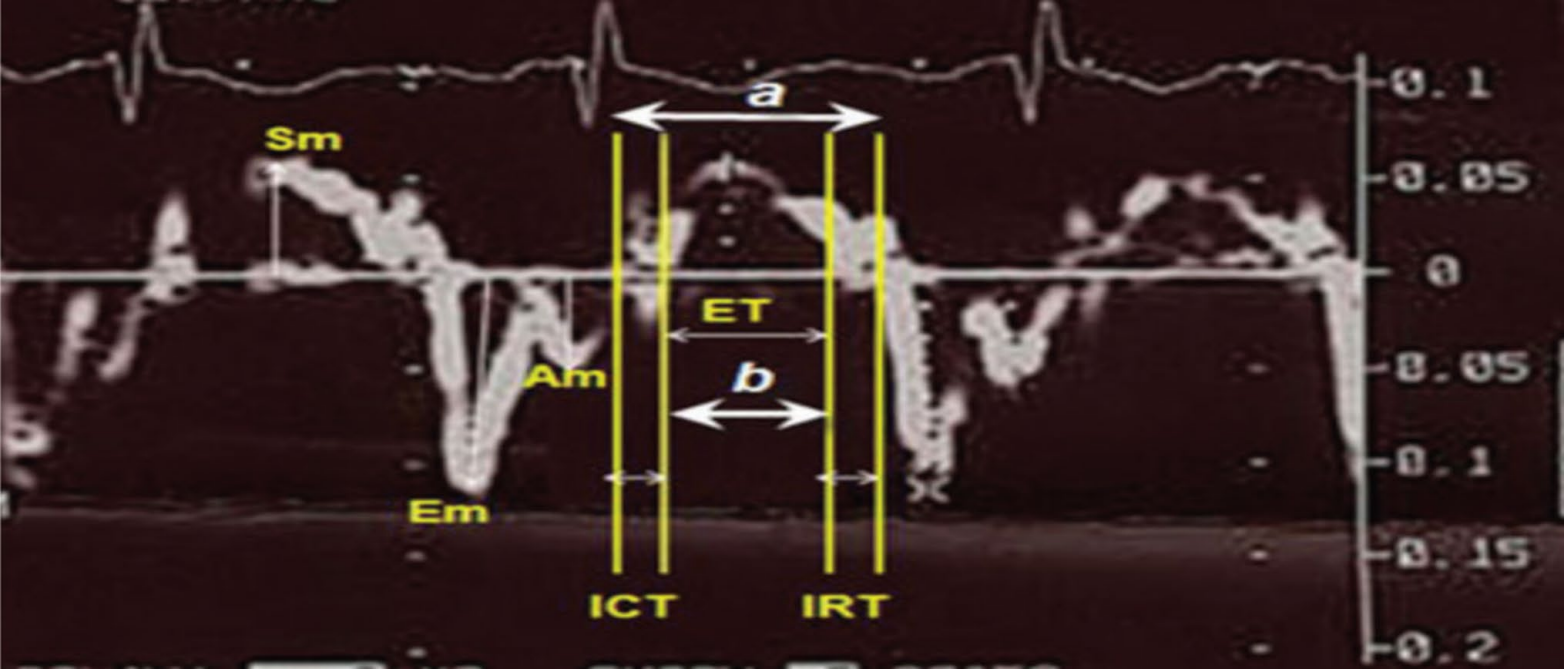
Example of calculation of left ventricular Doppler tissue imaging (DTI) measurements

The detailed DTI measurements are shown in Fig. 1. The interval (a) between the end of the late diastolic annular velocity and the onset of the early diastolic annular velocity was equal to the sum of the isovolumetric contraction time (ICT), isovolumetric relaxation time (IRT), and ejection time (ET). The ET (b) was measured as the duration of the systolic annular velocity (Sm). The sum of the ICT and IRT was obtained by subtracting (b) from (a). The IRT was measured from the pulsed‑wave Doppler tissue recordings as the time interval from the end of the systolic annular velocity to the onset of the early diastolic annular velocity, and the ICT was obtained by subtracting the IRT from (a − b). Then, the left ventricular (LV) Tei index was calculated as (a − b)/b. The ICT and ET are affected by systolic dysfunction, whereas the IRT is affected by diastolic dysfunction. Consequently, the Tei index is regarded as a valuable index for assessing global systolic and diastolic myocardial function at the same time.

### Myocardial strain

Myocardial strain parameters were assessed using 2-dimensional (2D) speckle tracking echocardiography image acquisition and offline analysis. We evaluated the LV peak and end-systolic global longitudinal strain (LVGLS): average of the basal septum, mid septum, apical septum, apical lateral wall, mid lateral wall, and basal lateral wall. Time to peak (TTP) systolic LVGLS (average of 6 segments) as well as for the septal and lateral walls (average of 3 segments) were calculated. All LV strain values are negative values, where a decrease in strain (hence, more positive value) is observed when LV function deteriorates.

### Statistical analysis

Data analysis was performed by SPSS software, version 30 (SPSS Inc., PASW Statistics for Windows version 30, Chicago: SPSS Inc.). Qualitative data are expressed as frequencies and percentages. Quantitative data were described using median (minimum and maximum) (interquartile range) for non-normally distributed data and mean ± standard deviation for normally distributed data after testing normality using the Shapiro–Wilk test. The significance of the obtained results was judged at the 0.05 level. The chi-square test was used to compare qualitative data between groups as appropriate. Mann–Whitney *U* was used to compare two studied groups for non-normally distributed data. Student’s *t*-test was used to compare two independent groups for normally distributed data.

## Results

### Electrocardiography

Based on the ECG, all patients and controls exhibited sinus rhythms with normal conduction durations (including PR interval, QRS interval, and QTc interval). No abnormalities in the ST segment were observed. Furthermore, there were no signs of ST-segment abnormalities nor were the ECG criteria for left or right ventricular hypertrophy met.

### Conventional echocardiography

II- Cardiac dimensions and function using conventional echocardiography in patients with NF1 and controls are listed in Table 1.

**Table 1:** Conventional echocardiographic measurements

Parameter	NF1 - patients (38)	Control(35)	*P*
FS%	38.22 ± 5.93	39.03 ± 5.43	0.5458
EF %	67.61 ± 8.15	73.22 ± 5.30	0.0009*
LVIDs (cm)	2.375 ± 0.54	2.249 ± 0.43	0.2765
LVIDd (cm)	3.79 ± 0.73	3.701 ± 0.62	0.5779
IVSs (cm)	0.87 ± 0.21	0.6 ± 0.05	< 0.0001*
IVSd (cm)	0.505 ± 0.08	0.48 ± 0.12	0.2950
LVPWs (cm)	0.593 ± 0.06	0.52 ± 0.03	< 0.0001*
LVPWd (cm)	0.47 ± 0.11	0.49 ± 0.13	0.4791
Mitral inflow E/A ratio	1.92 ± 0.29	1.88 ± 0.36	0.6014

*FS*, fractional shortening; *EF*, ejection fraction; *LVIDs*, left ventricular internal diameter at end systolic; *LVIDd*, left ventricular internal diameter at end diastolic; *IVSs*, inter-ventricular septal thickness at end systole; *IVSd*, inter-ventricular septal thickness at end diastole; *LVPWs*, left ventricular posterior wall thickness at end systole;* LVPWd*, left ventricular posterior wall thickness at end diastole; *E*, wave early filling wave velocity; A, wave atrial contractility wave velocity

*Considered significant as compared to the control group

As regards to the M-Mode echocardiographic measurements, the patients and the control children did not differ significantly as regard LVIDs, LVIDd, FS, IVSd, LVPWd, and Mitral inflow E/A ratio. But, with respect to controls, NF1 patients had significantly decreased EF and increased thickness of IVSs and LVPWs.

### Doppler tissue imaging (DTI) studies

The data from the DTI of both study groups are summarized in Table 2. Concerning the LV function by DTI echocardiography in the two groups, it was found that NF1 patients had significantly lower mitral peak systolic (Sm) wave velocity and lower early diastolic (Em) wave velocity.

**Table 2:** Doppler tissue imaging measurements

Parameter	NF1 patients (38)	Control (35)	*P*
Mitral annulus:			
*Mitral peak systolic (Sm) velocity (cm/s)*	8.06 ± 1.11	9.79 ± 0.89	< 0.0001*
*Mitral early diastolic (Em) velocity (cm/s)*	15.42 ± 2.40	17.9 ± 4.29	0.0029*
*Mitral late diastolic (Am) velocity (cm/s)*	8.67 ± 1.95	6.92 ± 1.69	0.0001*
*Mitral ICT (ms)*	58 ± 9.69	49.5 ± 4.1	< 0.0001*
*Mitral IRT (ms)*	56 ± 8.98	50.1 ± 3.23	0.0005*
*Mitral ET (ms)*	238.10 ± 34.93	330.1 ± 8.94	< 0.0001*
*Left ventricular Tei index:*	0.60 ± 0.15	0.32 ± 0.04	< 0.0001*

(Sm), systolic myocardial velocity; (Em), early myocardial diastolic velocity; (Am), late myocardial diastolic velocity; *ICT*, isovolumic contraction time; *IRT*, isovolumic relaxation time; *ET*, ejection time

*Considered significant as compared to the control group

As compared to control group, there was a significantly higher mean late diastolic (Am) velocity, significantly prolonged mitral ICT and IRT values, significantly shortened ET, and consequently higher LV Tei index values (*P* < 0.0001) in the NF1 group.

### Myocardial strain

2D speckle tracking-derived myocardial strain parameters of patients with NF1 and the controls are shown in Table 3.

**Table 3:** Two-dimensional speckle tracking-derived myocardial strain parameters of patients with NF1 and controls

Parameter	NF1 patients (38)	Control (35)	*P*
*Peak systolic LVGLS (%)*	− 20.41 ± 3.68	− 23.1 ± 3.18	0.0014*
*TTP systolic LVGLS (ms)*	289.49 ± 35.19	290.35 ± 38.12	0.9204
*Peak systolic septal WS (%)*	− 19.89 ± 4.18	− 22.69 ± 3.98	0.0046*
*TTP systolic septal WS (ms)*	276.68 ± 42.47	289.65 ± 32.61	0.1503
*Peak systolic lateral WS (%)*	− 19.93 ± 3.98	− 22.45 ± 2.79	0.0027*
*TTP systolic lateral WS (ms)*	277.56 ± 51.11	292.14 ± 35.78	0.1657

*LVGLS* left ventricular global longitudinal strain, *TTP* time to peak, *WS* wall strain

*Considered significant as compared to the control group

Peak systolic LVGLS, peak systolic septal, and lateral wall strain were the only significantly lower parameters in patients with NF1 compared to controls.

## Discussion

Neurofibromatosis type 1 (NF1) is an autosomal dominant syndrome with multisystem involvement; however, cardiac abnormalities are not included in the diagnostic criteria of NF1, and there are currently no clear guidelines for routine echocardiography evaluation in this population. Consequently, cardiac anomalies and functional impairments are likely underrecognized. In the present study, we evaluated cardiac status in 38 pediatric patients with NF1.

Our patients’ systolic and diastolic blood pressures were normal (adjusted for age) during their outpatient visits. So its potential impact on any detected cardiac abnormality was excluded.

Our study showed that NF1 children had statistically significantly increased thickness of IVSs and LVPWs compared to the control group, using M-Mode echocardiography.

This is consistent with a study conducted among 22 NF1 patients that revealed a significant increase in IVS thickness (*p*-value 0.001) compared to healthy subjects [16].

Although most research showed no decrease in EF in NF1 patients, one of these popular studies was conducted among 58 patients with NF1, which revealed normal EF [17]. Moreover, a study done among 22 NF1 patients showed normal EF [16]. However, our study showed a significant decrease in EF.

Using a non-invasive tool to assess systolic and diastolic function by calculating the Tei index with easily obtained echocardiographic Doppler tissue imaging parameters, we found that children with NF1 have significantly abnormal DTI parameters and consequently higher LV Tei index value (*P* < 0.0001), which reflects systolic and diastolic dysfunction in these patients as the normal value of Tei index is < 0.39 ± 0.05 [18].

This copes with a study done among 55 NF1 patients, which assessed cardiac function using Doppler tissue echocardiographic assessment that showed that all studied parameters of DTI were significantly different in patients with NF when compared with healthy subjects, suggesting that patients with NF1 may have abnormal systolic and diastolic function [19].

Myocardial strain measures are used in conjunction with traditional echocardiography to assess heart function [14]. Myocardial strain is a dimensionless indicator of total ventricular myocardial deformation during a cardiac cycle. Longitudinal strain is the dominant component of systolic strain [20].

Global longitudinal strain (GLS), which measures myocardial shortening of the left ventricular segments from base to apex, is the most reliable and clinically useful indicator for early detection of declines in cardiac function [21]. Compared to 2D echo, it correlates better with cardiac MRI (CMRI) [22]. GLS predicts cardiovascular events earlier and more accurately than LVEF [23].

Our study assessed myocardial strain using 2-dimensional (2D) speckle tracking echocardiography images and showed significantly lower peak systolic LVGLS, peak systolic septal, and lateral wall strain than control subjects, suggesting the presence of discrete myocardial dysfunction at the fiber muscle level. This copes with a study done among 58 NF1 patients, which assessed myocardial strain and revealed lower peak systolic left ventricular global circumferential strain and end systolic left ventricular global circumferential strain than control healthy subjects [17]. Additionally, another study conducted among 22 NF1 patients showed a lower global longitudinal strain than the control normal population [16].

The concomitant impairment of both ejection fraction (EF), left ventricular global longitudinal strain (LVGLS), and abnormally higher LV Tei index in our cohort may indicate the presence of functional hypertrophy rather than true structural remodeling. This interpretation is supported by the finding that the increased systolic thickness of the septal and posterior walls in NF1 patients was accompanied by diastolic wall dimensions comparable to those of the control group. Such functional hypertrophy likely reflects a compensatory adaptive response aimed at preserving overall myocardial contractile performance despite underlying subclinical myocardial involvement. This functional hypertrophy can progress to structural hypertrophy because of the progressive nature of the disease (as our study was conducted in the pediatric age group).

Additionally, the loss of myocardial neurofibromin in NF1 plays a vital role in the cardiovascular system. In the growing heart, it regulates the proliferation and epithelial-mesenchymal transformation [24]. Also, it is associated with activation of pathways known to be implicated in cardiac hypertrophy and dysfunction. One of the primary elements of intracellular signal transduction necessary for healthy cardiac growth is the Ras family of guanosine triphosphate (GTP)-binding proteins (G proteins), which is also crucial for the onset of ventricular hypertrophy and heart failure [15].

Moreover, neurofibromin was also discovered in the arterial wall [25], so vessel dysplasia, intimal growth, and elastic tissue fragmentation and consequently poor vascular compliance occur in NF1 [26]. These factors can all lead to systolic and diastolic dysfunction in these patients.

Although our study focused on asymptomatic NF1 individuals who had not received any specific therapy, it is important to note that the emerging treatment selumetinib, used for plexiform neurofibromas, has been reported to negatively impact myocardial function. Caiffa et al. demonstrated subtle yet measurable differences in global longitudinal strain (GLS) between selumetinib-treated NF1 patients (*n* = 17) and healthy controls [27].

This point is very important as our study identified a reduction in ejection fraction and LVGLS among NF1 children who did not receive any treatment, so a comprehensive baseline cardiac evaluation is strongly recommended before initiating Selumetinib.

## Strengths and limitations

Our study’s limitations include its small sample size and single-center design, but it is important to keep in mind that we are studying a relatively rare condition. Moreover, it was not assessed how heart function deteriorated over time.

So, future clinical outcomes studies should follow up on this cohort and enroll more patients to determine the impact of myocardial strain parameters on cardiovascular outcomes in pediatric NF1 patients.

One of the advantages is that our findings are attributable to the disease under investigation because all of the participants were young and free of cardiovascular risk factors or comorbidities. The application of more modern cardiology technologies, such as global longitudinal strain, which offers quantitative measurement and is independent of the operator, is another strength. Furthermore, echocardiography is a widely accessible, non-invasive, radiation-free, and minimally uncomfortable technology that is frequently utilized for the early diagnosis of cardiac diseases.

## Conclusion

Our findings show that NF1 children can have early changes in heart function, even without symptoms or disorders like hypertension, which is a typical cardiac consequence in neurofibromatosis. The exact cause of these changes is unknown; however, they may have a vascular component and also be due to myocardial neurofibromin loss in RASopathies, as mentioned before.

Furthermore, follow-up ECHO is crucial as NF1 is a progressive disease, and thicker IVS and LVPW may progress to hypertrophic cardiomyopathy. Also, follow-up BP is vital even in patients with normal blood pressure, as HTN may develop in the future.

## Data Availability

The data sets generated during and/or analyzed during the current study are available from the corresponding author on reasonable request.

## Abbreviations

2D: Two-dimensional
Am: Late diastolic myocardial velocity
BSA: Body surface area
CHD: Congenital heart disease
CMRI: Cardiac magnetic resonance imaging
DTI: Doppler tissue imaging
E/A ratio: Mitral inflow early-to-atrial filling velocity ratio
ECG: Electrocardiogram/electrocardiography
ECHO: Echocardiography
EF: Ejection fraction
Em: Early diastolic myocardial velocity
ET: Ejection time
FS: Fractional shortening
GTP: Guanosine triphosphate
HCM: Hypertrophic cardiomyopathy
HTN: Hypertension
ICT: Isovolumic contraction time
IRT: Isovolumic relaxation time
IVSd: Interventricular septal thickness at end diastole
IVSs: Interventricular septal thickness at end systole
LV: Left ventricle/left ventricular
LVGLS: Left ventricular global longitudinal strain
LVIDd: Left ventricular internal diameter at end diastole
LVIDs: Left ventricular internal diameter at end systole
LVPWd: Left ventricular posterior wall thickness at end diastole
LVPWs: Left ventricular posterior wall thickness at end systole
MUCH: Mansoura University Children’s Hospital
NF1: Neurofibromatosis type 1
RAS/MAPK: Rat sarcoma/mitogen-activated protein kinase
Sm: Systolic annular velocity
TTP: Time to peak
WS: Wall strain
